# *Lobatozoum woodi* n. sp. (Digenea: Didymozoidae) parasitizing *Euthynnus alletteratus* (Scombriformes: Scombridae) in the coast of the State of Rio de Janeiro, Brazil

**DOI:** 10.1590/S1984-29612024065

**Published:** 2024-11-04

**Authors:** Robertta Gitahy Freire, Marcia Cristina Nascimento Justo, Yuri Costa Meneses, Alena Mayo Iñiguez, Simone Chinicz Cohen

**Affiliations:** 1 Laboratório de Helmintos Parasitos de Peixes, Instituto Oswaldo Cruz, Fundação Oswaldo Cruz – IOC/FIOCRUZ, Rio de Janeiro, RJ, Brasil; 2 Programa de Pós-Graduação em Biodiversidade e Saúde, Instituto Oswaldo Cruz, Fundação Oswaldo Cruz – IOC/FIOCRUZ, Rio de Janeiro, RJ, Brasil; 3 Laboratório de Parasitologia Integrativa e Paleoparasitologia – LPIP, Instituto Oswaldo Cruz, Fundação Oswaldo Cruz – IOC/FIOCRUZ, Rio de Janeiro, RJ, Brasil

**Keywords:** Atlantic Ocean, didymozoid, new species, Trematoda, Oceano Atlântico, didimozoídeo, nova espécie, Trematoda

## Abstract

The aim of this study was to describe a new species of Didymozoidae (Trematoda) found in the mesentery of *Euthynnus alletteratus* (Rafinesque) off Cabo Frio, in the coastal area of the state of Rio de Janeiro, Brazil. Thirty specimens of *E. alletteratus* were obtained between August 2023 and June 2024, directly from traders who sell fresh fish in markets. The parasites were fixed in AFA (93% ethanol 70%, 5% formaldehyde and 2% glacial acetic acid) with or without compression, stained with Langeron’s hydrochloric carmine, dehydrated in an alcohol series, clarified in clove oil and mounted in Canada balsam as permanent slides. The new species was assigned to the genus *Lobatozoum* Ishii, 1935, mainly by the presence of lobes in the posterior region of the body and by the arrangement of the testes, ovary and vitellaria. *Lobatozoum woodi* n. sp. differs from all congeneric species mainly by presenting three testicular tubules, by the size of pharynx, larger than the oral sucker, and by the absence of digestive glands cells in esophagus and initial portion of caeca. The finding of a new species of *Lobatozoum* represents the eleventh valid species in the genus.

## Resumo

O objetivo deste estudo foi descrever uma nova espécie de Didymozoidae (Trematoda), encontrada no mesentério de *Euthynnus alletteratus* (Rafinesque), coletado em Cabo Frio, zona costeira do estado do Rio de Janeiro, Brasil. Trinta espécimes de *E. alletteratus* foram obtidos entre agosto de 2023 e junho de 2024, diretamente de comerciantes que comercializam peixe fresco em mercados. Os parasitos foram fixados em AFA (93% etanol 70%, 5% formaldeído, 2% ácido acético glacial) com ou sem compressão, corados com carmim clorídrico de Langeron, desidratados em série alcoólica, clarificados em óleo de cravo e montados em bálsamo do Canadá como lâminas permanentes. A nova espécie foi atribuída ao gênero *Lobatozoum* Ishii, 1935, principalmente devido à presença de lóbulos na região posterior do corpo e pelo arranjo dos testículos, ovário e vitelária. *Lobatozoum woodi* n. sp. difere de todas as espécies congêneres principalmente por apresentar três ramos testiculares, tamanho da faringe, que é muito maior que a ventosa oral e ausência de células glandulares no esôfago e porção inicial do ceco. *Lobatozoum woodi* n. sp. representa a décima primeira espécie válida do gênero.

## Introduction

Scombrids (tuna, bonitos, Spanish mackerels and mackerels) are important fishery resources distributed in tropical, subtropical and temperate waters and are among the most important marine resources in the world. These fishes stand out economically through their use for human consumption purposes and socially as a form of sports practice (Juan-Jordá et al., 2013; FAO, 2020).

Scombrid fishes have a high capture rate, which is explained by their characteristic of forming schools, consequently favoring the fishing industry (Contreras-Guzmán, 1994). The species belonging to the Thunnini tribe include bonitos and tunas, which are epipelagic and mesopelagic species that have a high migratory capacity (Fagundes et al., 2000).

*Euthynnus alletteratus* (Rafinesque) (Scombriformes: Scombridae), popularly known in Brazil as “bonito-pintado”, is a species that has coastal habits, with preferences for surface waters of tropical and subtropical regions, including the Mediterranean, Black Sea, Caribbean Sea and Gulf of Mexico. These fish are opportunistic predators that feed on practically everything within its range, i.e. crustaceans, fishes, squids, heteropods, and tunicates (Froese & Pauly, 2024). According to Madhavi & Sai Ram (2000), the characteristics of *E. alletteratus*, such as high vagility, varied diet, long life span, and endothermy caused by the presence of a countercurrent heat exchange system, make this host more susceptible to helminth parasites.

Trematodes belonging to Didymozoidae Monticelli, 1888 stand out among the Platyhelminthes that parasitize *E. alletteratus*. They are usually found encapsulated in pairs in the tissues of marine teleosts, mainly those of the Scombridae (Pozdnyakov & Gibson, 2008). Species of Didymozoidae have been referred in *E. alletteratus*: *Allopseudocolocyntotrema alioshkini* Pozdnyakov, 1994, *Coeliotrema thynni* Yamaguti, 1938, *Didymocodium euthynni* Yamaguti, 1970, *Didymocystis exiguus* (Yamaguti, 1970) Pozdnyakov, 1990, *Didymocystis reniformis* Ariola, 1902, *Koellikerioides intestinalis* Yamaguti, 1970, *Lobatozoum euthynni* (Ching & Madhavi, 1999), *Lobatozoum multisacculatum* Ishii, 1935, *Melanocystis kawakawa* Yamaguti, 1970, *Neonematobothrium annakohnae* Justo, Cardenas & Cohen, 2021, *Oesophagocystis lydiae* Pozdnyakov, 1994, *Phacelotrema claviforme* Yamaguti, 1951 and *Pseudocolocyntotrema yaito* Yamaguti, 1970 (Alves & Luque, 2006; Justo & Kohn, 2009, 2011; Justo et al., 2020, 2021; Mele et al., 2016; Pozdnyakov, 1994, 1996; Yamaguti, 1938).

Didymozoidae includes greatly different morphological genera. *Lobatozoum* Ishii, 1935 (Didymozoinae) is characterized by body shape, which presents the posterior region much widened, diverse in shape, with numerous regularly or irregularly alternating protrusions, indentations or lobes; pharynx present; esophagus surrounded with or without gland cells; testes cylindrical, long, rarely branched; ovary and vitellarium branched (Pozdnyakov & Gibson, 2008). The genus comprises ten valid species: *L. arielii* (Nikolaeva & Dubina, 1978) Pozdnyakov, 1989; *L. bengalense* (Hussain, Hanumantha Rao & Shyamasundari, 1985) Pozdnyakov, 1996; *L. bilobatum* Hyman, 1963; *L. euthynni*; *L. kawakawa* (Yamaguti, 1970) Pozdnyakov, 1989; *L. macrolobulare* Pozdnyakov, 1989; *L. multisacculatum*; *L. sinicum* (Shen, 1984) Pozdnyakov, 1996; *L. yaito* (Yamaguti, 1965) Pozdnyakov, 1989 and *L. yamagutii* (Madhavi, 1982) (WoRMS, 2024).

During research on parasites of scombrids from the coast of the state of Rio de Janeiro, a new species of *Lobatozoum* was found in *E. alletteratus*, which is described in the present report.

## Material and Methods

A total of 30 specimens of *E. alletteratus* (31 to 67 cm in total length and 430 g to 5,300 kg in weight) collected off the coast of the state of Rio de Janeiro (22º52’46’ S, 42º01’07” W), were obtained from local fishermen in Cabo Frio, Rio de Janeiro, Brazil, and in the municipal market of São Pedro in Niteroi, Rio de Janeiro, Brazil, between August 2023 and June 2024. The fish were kept refrigerated and carried to the Laboratory of Helminth Parasites of Fishes, Oswaldo Cruz Institute, Fiocruz, Rio de Janeiro, Brazil, to be examined. Capsules containing parasites were carefully removed from the tissue and dissected, using needles to release the parasites. The didymozoids were fixed in AFA (93% ethanol 70%, 5% formaldehyde and 2% glacial acetic acid) under slight coverslip pressure between two glass slides or without pressure, stained with Langeron's hydrochloric carmine, dehydrated in an alcohol series, cleared in clove oil and mounted in Canada balsam as permanent slides.

The specimens were studied using a Zeiss Axioskop® 2 microscope, which is equipped with a drawing tube and digital camera that were used to make illustrations and photomicrographs. Observations were also made using a Zeiss LSM 980® laser scanning confocal microscope equipped with Airyscan 2, to better visualize the internal structures. The measurements are expressed in micrometers (µm) except where indicated. Ranges are followed by the average in parentheses and the number of samples measured in square brackets.

The holotype and paratypes were deposited in the Helminthological Collection of the Oswaldo Cruz Institute (CHIOC), Rio de Janeiro, Brazil.

## Results

Thirty specimens of *E. alletteratus* were examined for the presence of didymozoid parasites and 28 yellow parasites encapsulated in pairs in the mesentery were found in one fish.

Class Trematoda Rudolphi, 1808

Subclass Digenea Carus, 1863

Order Plagiorchiida La Rue, 1957

Superfamily Hemiuroidea Looss, 1899

Family Didymozoidae Monticelli, 1888

Subfamily Didymozoinae Monticelli, 1888

*Lobatozoum* Ishii, 1935

**Figure 1:**
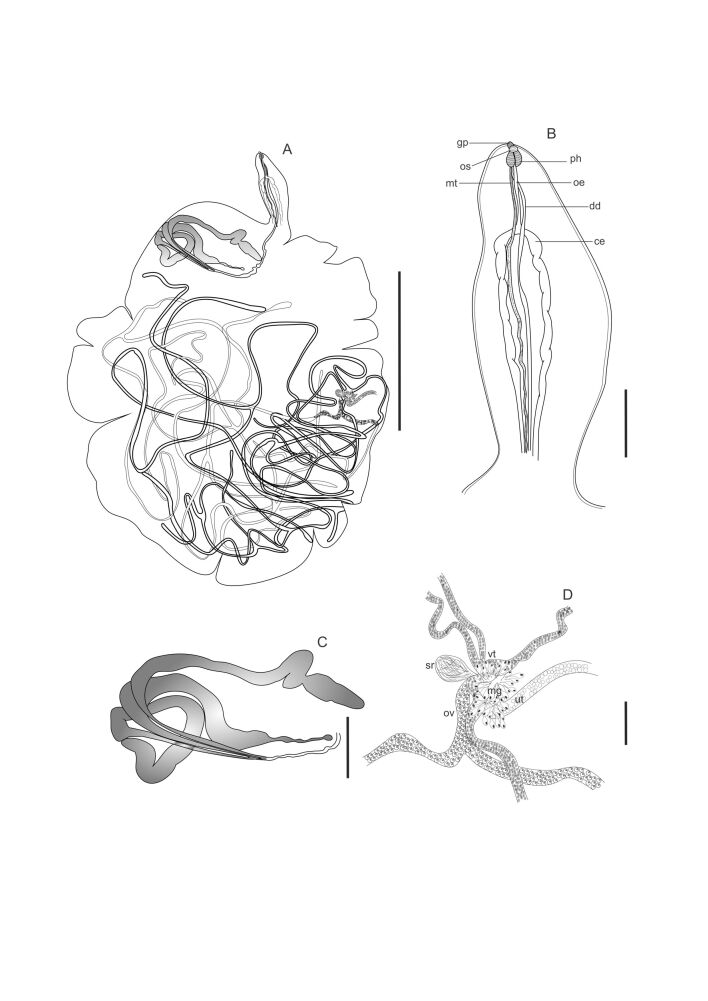
*Lobatozoum woodi* n. sp. from *Euthynnus alletteratus*. **A** - Total view (branches with darker lines represent the vitellaria, and branches with lighter lines represent the ovary). **B** - Anterior region of the body, showing the oesophagus (oe), caeca (ce), oral sucker (os); pharynx (ph), ductus deferens (dd), and metraterm (mt) uniting at the level of genital pore to form a short hermaphroditic duct that opens via a well-developed genital pore (gp). **C** – Testes. **D**. Genital junction showing detail of the tubular ovary consists of a short system forming two main branches (ov); vitellaria formed by 4 main branches (vt); well-developed Mehlis’s gland (mg), uterus (ut) and seminal receptacle (sr). Bars: A= 2 mm; B= 150 µm, C= 50 µm; D= 200 µm.

**Figure 2:**
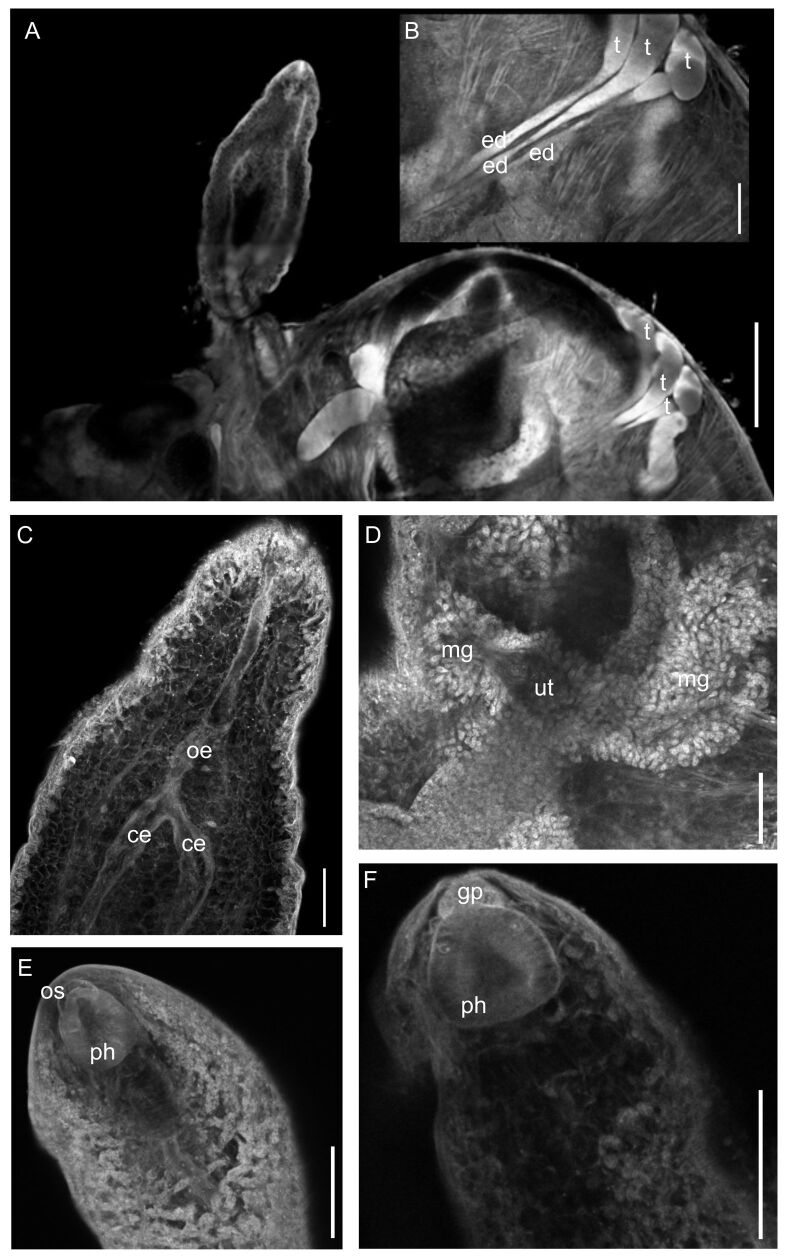
*Lobatozoum woodi* n. sp. confocal laser scanning micrographs. A - Anterior part of the posterior region of the body, showing the location of the testes (t). **B** - Detail of the testes (t) showing the efferent ducts (ed). **C** - Oesophagus (oe); caeca (ce). **D** - Detail of the well-developed Mehlis’s gland (mg) and uterus (ut). **E**- Pharynx (ph) much larger than the oral sucker (os). **F** - Pharynx (ph) and genital pore (gp). Bars: A, B = 500 µm; C, E, F = 100 µm; D = 50 µm.

**Figure 3:**
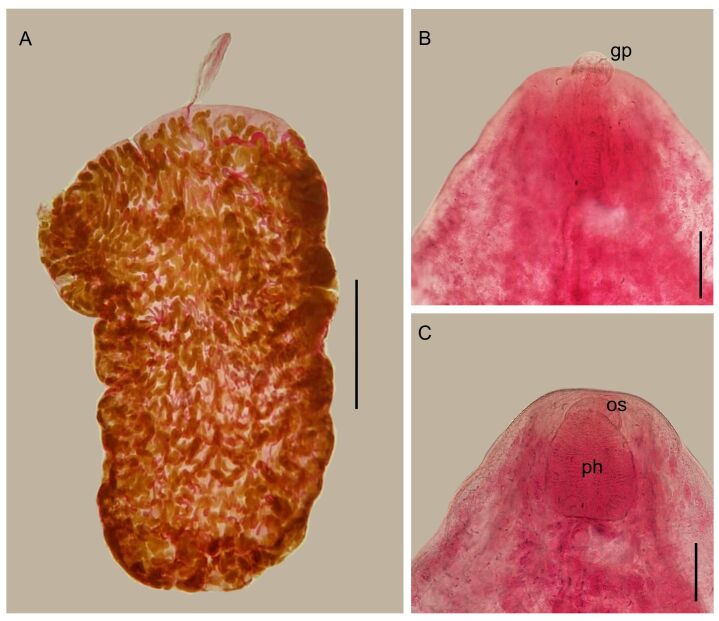
*Lobatozoum woodi* n. sp. Light photomicrographs. **A** - Total view. **B** - Anterior region of the body, showing well-developed genital pore (gp). **C** - Pharynx (ph) and oral sucker (os). Bars: A=1.5 mm; B, C=50 µm.

***Lobatozoum woodi* n. sp**. (Figures 1-3)

urn:lsid:zoobank.org:pub:2AD67CEC-2BB9-4A32-B10A-92B96CE0A0EE

Type-host: *Euthynnus alletteratus* (Rafinesque, 1810) (Scombriformes, Scombridae).

Type-locality: Cabo Frio, Rio de Janeiro, Brazil.

Site of infection: mesentery.

Parasitological indexes: Total number of hosts: 30; number of infected hosts: 1; total number of parasites: 28.

Specimens deposited: Holotype CHIOC 40445 a; Paratypes CHIOC 40445 b-g.

Etymology: The specific name is an honor to the veterinarian Woodson Leira Cordeiro, who was affectionately called “Wood” by his family and friends, and was the fiancé of the first author, but passed away at a young age on April 22, 2023. Dr. Woodson Cordeiro was a great enthusiast on the area of food supply in veterinary medicine.

Description: Based on 12 adult specimens, stained with Langeron's hydrochloric carmine: Yellow capsules with two hermaphroditic individuals with identical morphology, easily visible to the naked eye. Tegument thin and delicate. Body divided into two regions (Figures 1A; 3A). Anterior region elongated, subcylindrical, 0.85–3.0 (1.87) mm [n = 8] long by 0.30–0.64 (0.48) mm [n = 7] wide, at the level of the esophagus bifurcation (Figures 1B; 2A; 3A). Oral sucker inconspicuous, subterminal, non-muscular, 28–48 (37) [n = 7] long by 30–63 (48) [n = 7] wide (Figures 1B; 2E; 3C); pharynx strongly muscular, larger than oral sucker, 68–132 (92) [n= 9] long by 48–95 (67) [n = 9] wide (Figure 1B; 2E,F; 3C); esophagus straight to slightly sinuous, narrow, without digestive glands, 0.25–0.48 (0.37) mm [n = 7] long (Figures 1B; 2C); intestine bifurcated into two caeca in anterior region (Figures 1B; 2C), not visible in the posterior region; caeca without digestive glands in the initial portion. Sinuous duct deferens, entering the anterior region together with the metraterm, joining at the level of the pharynx to form the hermaphroditic duct (Figure 1B), ending in a prominent genital pore, ventral to the base of the oral sucker (Figures 1B; 2F; 3B). Posterior region semi-oval in shape, with several pronounced lateral lobulations on both sides, 2.95–11.95 (5.69) mm [n = 12] long by 2.84–7.20 (3.90) mm [n = 12] maximum width (Figures 1A; 3A). Testes divided into 3 long tubules, concentrated at one of the sides in the initial part of the posterior region of body, 2.43–3.02 (2.64) mm [n = 3] long (Figures 1A,C; 2A,B); duct deferens sinuous, running along with metraterm, opening at a prominent genital pore dorsal to the base of the oral sucker. Ovary tubular, consisting of a short system forming two main branches, each of which divides into several other very slender terminal branches (Figure 1D); vitellaria formed by 4 main branches that divide into several terminal branches (Figure 1D). Number of ovary and vitelline branches not defined because they are intertwined with countless uterine loops full of eggs. Genital junction located in the middle of the posterior region of the body, close to the lateral edge. Mehlis’ gland well-developed (Figures 1D; 2D). Seminal receptacle rounded 200 [n = 1] (Figure 1D). Morphology of the uterus characteristic of the family, spread across the posterior region of the body, strongly distended by the massive presence of eggs (Figures 1D, 2D). Egg reservoir in the anterior portion of the posterior region. Eggs operculated, 12–13 (12) [n = 44] long by 7–8 (8) [n = 44] wide.

**Remarks**: The new species is allocated in *Lobatozoum* by presenting the characteristics of the genus, mainly the body shape, which presents the posterior region much widened, with numerous irregular lobes. *Lobatozoum woodi* n. sp. differs from all congeneric species mainly in terms of the number of testicular tubules. The new species is most closely related to *L. euthynni*, *L. bengalense* and *L. yaito* mainly by presenting more than two tubular testes, while other species present only two tubular testes. However, the new species, in which the testes are formed by 3 long unbranched tubules, differs from these species in the number of tubules, which in *L. euthynni* and *L. yaito* range from 7 to 8 unbranched tubules, while in *L. bengalense* they are composed of 8 thin and strongly coiled tubules. The new species also differs from these three in terms of the location of the testes, restricted to one side of the anterior portion of the posterior region of the body in the new species, while in the other three they are located centrally at the base of the junction with the anterior region.

*Lobatozoum woodi* n. sp. also differs from *L. yaito* and *L. euthynni* in terms of the shape and size of the oral sucker, which is weakly muscular and small (28–48 x 30–63 µm) in the new species, whereas in *L. yaito* it is much larger (110–160 x 120–180 µm) and in *L. euthynni* it is larger and divided into a muscular and a non-muscular region (164–219 x 90–100 µm). The pharynx of *L. woodi* n. sp. also differs from these two species in size, such that it is smaller (69–132 x 48–95 µm) than that of *L. yaito* (140–200 x 130–200 µm) and larger than that of *L. euthynni*, in which it is a rudimentary organ.

## Discussion

*Lobatozoum* was originally described by Ishii (1935) to accommodate the type species *Lobatozoum multisacculatum*, which was found encapsulated in the gills of *Thunnus orientalis* (Temminck & Schlegel) and *Katsuwonus pelamis* (Linnaeus) (= *Katsuwonus vagans* Lesson) from Japan.

Pozdnyakov (1989, 1996) and Pozdnyakov & Gibson (2008) considered *Lobatocystis* Yamaguti, 1965; *Kamegaia* Yamaguti, 1970; *Yamaguticystis* Nikolaeva & Dubina, 1978; *Lepidodidymozoon* Shen, 1984 and *Renodidymocystis* Madhavi, 1982 as synonyms of *Lobatozoum*, due to similarities that include the formation of lobes in the posterior region. However, Madhavi & Bray (2018) revalidated *Renodidymocystis*, considering that the lobed nature of the body, the occurrence of a single spirally twisted testis, and the absence of a seminal receptacle are distinct characteristics of *R. yamagutii*, the only species recorded in this genus so far. In this same paper, the authors refer *L. bengalense* and *L. yaito*, without mentioning the previous synonymies. According to WoRMS (2024), the above genera are considered synonyms of *Lobatozoum* and until now, 10 species are valid and 25 years later, the eleventh species of this genus is described in the western South Atlantic. *Lobatozoum woodi* n. sp. was found encapsulated in pairs in the mesentery of *E. alletteratus*, a site of infection that had not previously been reported for this genus, since most species were found in the gills (*L. arielii*, *L. bengalense*, *L. bilobatum*, *L. euthynni*, *L. macrolobulare*, *L. multisacculatum*, *L. yaito*), in the kidneys (*L. yamagutii*), in the intestine (*L. kawakawa*) and in the tegument under the scales (*L. sinicum*). Members of *Lobatozoum* parasitize mainly Scombridae (9 species), followed by Epinephelidae (1 species) and by Sphyraenidae (1 species), in the Indian Ocean, Pacific Ocean, China Sea and North Atlantic, and now recorded in the western South Atlantic.

Although Brazil is one of the largest tuna traders, most species of Didymozoidae have been described and reported in hosts mainly in the Pacific Ocean, Indian Ocean, and Mediterranean Sea (see Pozdnyakov & Gibson, 2008). However, since 2005, great efforts have been made to understand the diversity of didymozoids in the western South Atlantic and to date, 41 species of Didymozoidae have been reported, mainly in scombrid fishes in this area of the Atlantic Ocean (Justo & Kohn, 2014; Moreira-Silva et al., 2019; Justo et al., 2021) and, in the present paper, the diversity of this family is increased to 42 species.
